# YouTube as a Source of Patient Information on External Cephalic Version: Cross-Sectional Study

**DOI:** 10.2196/50087

**Published:** 2024-06-06

**Authors:** Merle R van Dijk, Anne-Fleur van der Marel, Leonie E van Rheenen-Flach, Wessel Ganzevoort, Etelka Moll, Fedde Scheele, Joost Velzel

**Affiliations:** 1 Department of Obstetrics and Gynecology Onze Lieve Vrouwen Gasthuis Amsterdam Netherlands; 2 Department of Obstetrics and Gynecology Amsterdam University Medical Centre Amsterdam Netherlands; 3 Amsterdam Reproduction & Development Research Institute Amsterdam Netherlands; 4 Faculty of Science Athena Institute Vrije Universiteit Amsterdam Netherlands; 5 Department of Obstetrics and Gynecology Northwest Clinics Alkmaar Netherlands

**Keywords:** YouTube, ECV, external cephalic version, breech, education, video, cesarean, health education, childbirth, patient information, cross-sectional study, cesarean delivery, implementation, usefulness, medical information, pregnancy, pregnant women, engagement

## Abstract

**Background:**

With the global increase of cesarean deliveries, breech presentation is the third indication for elective cesarean delivery. Implementation of external cephalic version (ECV), in which the position of the baby is manipulated externally to prevent breech presentation at term, remains suboptimal. Increasing knowledge for caretakers and patients is beneficial in the uptake of ECV implementation. In recent decades, the internet has become the most important source of information for both patients and health care professionals. However, the use and availability of the internet also bring about concerns since the information is often not regulated or reviewed. Information needs to be understandable, correct, and easily obtainable for the patient. Owing to its global reach, YouTube has great potential to both hinder and support spreading medical information and can therefore be used as a tool for shared decision-making.

**Objective:**

The objective of this study was to investigate the available information on YouTube about ECV and assess the quality and usefulness of the information in the videos.

**Methods:**

A YouTube search was performed with five search terms and the first 35 results were selected for analysis. A quality assessment scale was developed to quantify the accuracy of medical information of each video. The main outcome measure was the usefulness score, dividing the videos into useful, slightly useful, and not useful categories. The source of upload was divided into five subcategories and two broad categories of medical or nonmedical. Secondary outcomes included audience engagement, misinformation, and encouraging or discouraging ECV.

**Results:**

Among the 70 videos, only 14% (n=10) were defined as useful. Every useful video was uploaded by educational channels or health care professionals and 80% (8/10) were derived from a medical source. Over half of the not useful videos were uploaded by birth attendants and vloggers. Videos uploaded by birth attendants scored the highest on audience engagement. The presence of misinformation was low across all groups. Two-thirds of the vloggers encouraged ECV to their viewers.

**Conclusions:**

A minor percentage of videos about ECV on YouTube are considered useful. Vloggers often encourage their audience to opt for ECV. Videos with higher audience engagement had a lower usefulness score compared to videos with lower audience engagement. Sources from medically accurate videos should cooperate with sources with high audience engagement to contribute to the uptake of ECV by creating more awareness and a positive attitude of the procedure, thereby lowering the chance for a cesarean delivery due to breech presentation at term.

## Introduction

Breech presentation occurs in 3%-4% of singleton full-term pregnancies, and there remains controversy about the preferred mode of delivery in the clinical community [1,2]. Planned cesarean delivery (CD) in cases of breech presentation leads to a reduction in perinatal morbidity compared to vaginal breech delivery [3]. However, CD is a high-risk procedure associated with an increase in maternal morbidity and possible complications in future pregnancies [4]. Vaginal breech delivery remains common in many contexts and is considered a high-risk obstetrics procedure.

External cephalic version (ECV) is a medical intervention involving the application of abdominal pressure by health care professionals to facilitate the external rotation of a fetus from a breech presentation to a cephalic presentation. ECV is generally considered a safe procedure, associated with a low incidence of complications, and can effectively avert the need for a CD. ECV has been shown to reduce the incidence of breech presentation at term, resulting in less high-risk vaginal deliveries and CD for breech presentation [5]. The number of ECV procedures necessary to prevent one CD is estimated to be 2.6 (95% CI 2.0-3.9), with a success rate of approximately 50% [6,7]. With the global increase of CD, breech presentation is the third indication for elective CD [8]. However, implementation of ECV to prevent breech presentation at term remains suboptimal, since not all caretakers are providing ECV and not all patients opt for the offered attempt [9]. Increasing knowledge for caretakers and patients would be beneficial in the uptake of the implementation.

In recent decades, a change has become evident in how patients and their communities obtain information on health-related subjects [10]. Currently, the internet is the most important source of information for both patients and health care professionals. Specifically, social medial plays a key role as a source of health information [11]. YouTube, as the most popular video-sharing website with over 5 billion daily views, includes an expanding library of health education videos [12]. However, the use and availability of YouTube are also associated with concerns since the content is not regulated or reviewed.

For patients, gathering correct information to come to a well-informed choice is important. This information needs to be understandable, accurate, and easily obtainable for the patient, and YouTube shows great potential in this regard. Studies have shown that videos posted on YouTube wield a profound influence on young adults by shaping and influencing their preferences, behaviors, and perceptions, becoming a primary source of information, entertainment, and cultural engagement in their lives [13-15]. Because of its global reach, YouTube has high potential to both hinder and support spreading medical information and can therefore be used as a tool for shared decision-making [16].

The quality of patient-education videos on YouTube has been studied in the context of several obstetric aspects, including CD, pain management after CD, abnormally invasive placenta, and medication use during pregnancy [17-21]. However, no study has yet evaluated the quality and content of YouTube videos on ECV. Therefore, in this study, we aimed to evaluate the accuracy of medical information about ECV available on YouTube.

## Methods

### Search Strategy

A systematic search of videos uploaded on YouTube was performed in March 2022 with the following five search terms: “external cephalic version,” “ECV,” “fetal version,” “turning a breech baby,” and “turning baby.” The search was performed using the Google Chrome browser operated in Incognito mode and no personal YouTube or Google account was used. Search results were sorted by relevance, replicating a default YouTube search. The first 35 results were collected per search term, since 90% of YouTube users do not watch videos past the 30th resulting video [22]. Inclusion criteria were (1) videos related to cephalic version, (2) English language, and (3) acceptable audio-visual quality. Exclusion criteria were (1) length>1 hour, (2) irrelevant videos, and (3) duplicates. Videos were evaluated by two authors (MRvD and AFvdM), representing the demographic of the viewers. At the time of reviewing the videos, MRvD was a PhD candidate with 1 year of clinical experience in gynecology and obstetrics and AFvdM was a medical student in her final year. Discrepancies were discussed with authors LEvRF and JV, who are both obstetricians.

### Video Assessment and Outcome Scores

For each video, the following quantitative data were collected: day of upload, years since upload, duration of the video in minutes, number of views, total number of likes and dislikes, and uploader’s username. To assess audience engagement, the like ratio ([likes× 100]/[likes+dislikes]), view ratio (number of views/days on platform), and video power index (VPI) ([like ratio×view ratio]/100) were determined for each video.

### Usefulness Score

#### Score Components and Calculation

Since there are no established assessment tools for evaluating the accuracy of medical information on videos about ECV, a self-developed usefulness score instrument was used. The usefulness score consisted of three parts: the ECV Royal College of Obstetricians & Gynaecologists (RCOG)–specific score [5,23], modified DISCERN (mDISCERN) score [24], and presence of misinformation. The total usefulness score was then calculated as the sum of the ECV RCOG and mDISCERN scores with points subtracted for misinformation. A maximum of 30 points could be rewarded to every video. The total usefulness score was used to categorize videos as not useful (<9 points), slightly useful (9-13 points), and useful (>13 points). Every video with a discrepancy of 4 points or more in the usefulness score between the two reviewers was reassessed. For the analyses, the average score of the two reviewers was used. The items and scoring scheme for the ECV RCOG–specific score and mDISCERN score are shown in Table 1.

**Table 1:** Components of the usefulness score instrument, a self-developed tool to evaluate the accuracy of medical information on videos about external cephalic version (ECV).

Usefulness score instrument^a^				Not mentioned		Mentioned		Clearly explained
**ECV RCOG^b^–specific score**								
	**General information**							
		Turning the baby by putting pressure on the pregnant person’s abdomen	0		1		2	
		Usually performed after 36 weeks of pregnancy	0		1		2	
		If Rhesus D–negative as a pregnant person, advise to have an anti-D injection after ECV	0		1		2	
		Goal of turning the baby is to aim for a vaginal birth	0		1		2	
	**Complications and exclusions**							
		Safe and very low complication rate, usually going home on the same day	0		1		2	
		Slightly higher risk of needing a vacuum, forceps, or emergency cesarean section when going into labor	0		1		2	
		Exclusion criteria for ECV: needing a cesarean section for other reasons, including recent vaginal bleeding, abnormal cardiotography (heart rate tracing of baby), ruptured membranes/broken water, and multiple pregnancy	0		1		2	
	**Outcome of ECV**							
		Chance of succeeding	0		1		2	
		Successful for approximately 50% of pregnant people	0		1		2	
		Possible to have a second attempt at ECV on a different day	0		1		2	
		In less than 5% of cases, a baby will turn back to breech position after a successful ECV	0		1		2	
**mDISCERN^c^**								
	Are the aims clear and achieved?		0		1		2	
	Are reliable sources of information used? (ie, publication cited, speaker is an epidemiologist or medical professional)		0		1		2	
	Is the information presented balanced and unbiased?		0		1		2	
	Are additional sources of information listed for patient reference?		0		1		2	

^a^A maximum of 30 points could be rewarded to every video. The total score is derived as the sum of the scores from all components of both scoring systems and then subtracting points deducted for misinformation; the total score was used to categorize videos as not useful (<9 points), slightly useful (9-13 points), and useful (>13 points).

^b^RCOG: Royal College of Obstetricians & Gynecologists.

^c^mDISCERN: modified DISCERN score; a validated tool that measures the reliability of patient information concerning treatment choice [24]. The modification was used to prevent overlap with certain questions in the RCOG-based score.

#### RCOG-Based Score

The ECV RCOG–specific score is based on the patient information web page of the RCOG No. 20a [5,23]. The informational pamphlet issued by the RCOG provides comprehensive details essential for individuals to make informed decisions and provide informed consent for undergoing this medical procedure. Each video could score a maximum of 20 points on the ECV RCOG–specific score, depending on information being not mentioned (0 points), mentioned (1 point), or clearly explained (2 points).

#### mDISCERN Score

The quality and reliability of each video was assessed by a modification of the DISCERN score, a validated tool that measures the reliability of patient information concerning treatment choices [24]. We used the mDISCERN to avoid redundancy with many items in the ECV RCOG–specific score and the original DISCERN tool. The mDISCERN score is based on 4 questions with a scale rated from 0 to 2, where 0 points were given if criteria were unfulfilled and 2 points were given if criteria were entirely fulfilled. A maximum of 8 points could be granted to each video.

#### Presence of Misinformation

Points could be deducted from the total usefulness score if either proven or unproven misinformation was given about the procedure, risks, pain, success rate, or the chance of the baby turning back in breech position. Unproven misinformation included videos mentioning incorrect or nonresearched success rates to turn a baby into a cephalic position by, for instance, postural management. With 2 points deducted in each case, the presence of misinformation could deduct a total of 16 points from the total usefulness score.

### Ethical Considerations

All videos in this study are publicly available on YouTube and all analyses were performed on deidentified data. This study is an observational study in which no action or behavior was imposed on participants. Therefore, this study is not subject to the Dutch Medical Scientific Research Involving Human Subjects Act [25].

### Video Classification

The primary outcome of the evaluation was the usefulness score. We performed several secondary analyses and report the scores obtained for prespecified groups. First, videos were categorized according to the source of upload into childbirth attendants, paramedics, vloggers, health care professionals, educational channels, and other. Second, videos were divided into medical videos, which included health care professionals, paramedics, and partly educational channels, and nonmedical videos, which included vloggers, childbirth attendants, and partly educational channels. Videos classified by educational channels were categorized according to the presenter’s profession. Third, all videos uploaded by vloggers were evaluated according to whether ECV was encouraged or discouraged.

### Statistical Analysis

The intraclass correlation was used to measure the reliability between the two reviewers with regard to the total usefulness score. The intraclass correlation is used to assess the consistency of measurements between raters. Among the multiple models available for this assessment, we selected the two-way mixed-effect model since we wanted to determine the level of agreement between the two raters. According to Koo and Li [26], based on the 95% CI, a value below 0.50 indicates poor correlation and a value above 0.90 indicates excellent correlation. Continuous variables are presented as mean (SD) and categorical variables are presented as n (%). For comparisons between groups, the χ^2^, Kruskal-Wallis, and Mann-Whitney *U* tests were used as appropriate. SPSS (version 28) was used for the analysis, where *P*<.05 was considered statistically significant.

## Results

### Video Selection

On March 16, 2022, 175 videos were selected from YouTube. Multimedia Appendix 1 shows a flowchart of the selection process of the included videos. After removing duplicates and applying the exclusion criteria, 70 videos remained for evaluation. Multimedia Appendix 2 provides an overview of all included videos. The mean duration of all videos was 8.9 minutes (SD 9.3) and the mean VPI was 203.8 (SD 449.8).

### Usefulness Score

The mean total usefulness score was 7.6 (SD 5.6). The intraclass correlation coefficient of the usefulness score between the two reviewers was 0.942, indicating excellent agreement [26]. Videos were categorized into useful (14.3%), slightly useful (22.9%), and not useful (61.4%) (Table 2). Videos scored as not useful had the highest audience engagement and videos scored as useful had the lowest audience engagement.

Over 60% of the videos defined as *not useful* were uploaded by vloggers and childbirth attendants. Most videos included in this category were found with the search term “turning a breech baby.” The majority of the videos defined as *slightly useful* were uploaded by educational channels and health care professionals (37.5% and 18.8%, respectively). All videos defined as *useful* were uploaded by educational channels and health care professionals, with 90% of the videos found with the search term “external cephalic version.”

**Table 2:** Characteristics of videos on YouTube about external cephalic version (ECV) divided by usefulness category and the source of upload or search term.

Category			Useful (n=10, 14.3%)		Slightly useful (n=16, 22.9%)		Not useful (n=43, 61.4%)		*P* value
Years since upload, mean (SD)			3.6 (3.4)		4.9 (3.2)		4.8 (3.2)		—^a^
Duration (minutes), (mean, SD)			12.3 (11.9)		9.3 (7.4)		7.0 (5.5)		—
**Audience engagement, mean (SD)**									
	View ratio^b^	49.8 (113.4)		246.2 (548.1)		261.9 (567.1)		.09	
	Like ratio^c^	82.1 (28.4)		89.6 (9.6)		92.1 (7.1)		.74	
	Video power index^d^	43.3 (95.5)		203.0 (430.9)		249.3 (502.4)		.10	
**Usefulness score^e^, mean (SD)**									
	Total score	17.9 (3.9)		11.0 (1.6)		3.9 (8.0)		<.001	
	ECV RCOG^f^ score points awarded	12.4 (3.2)		6.6 (1.7)		2.4 (2.1)		<.001	
	mDISCERN^g^ points awarded	6.0 (1.4)		5.0 (1.7)		2.2 (1.3)		<.001	
	Points deducted for misinformation	0.5 (1.0)		0.6 (0.9)		0.7 (1.0)		.57	
**Source of upload, n (%)**									
	Health care professional	4 (40.0)		3 (18.8)		5 (11.6)		—	
	Paramedic	0 (0)		2 (12.5)		4 (9.0)		—	
	Vlogger	0 (0)		1 (6.3)		17 (39.5)		—	
	Education channel	6 (60.0)		6 (37.5)		4 (9.3)		—	
	Childbirth attendant	0 (0)		1 (6.3)		11 (25.6)		—	
	Other	0 (0)		1 (6.3)		2 (4.7)		—	
**Search term, n (%)**									
	External cephalic version	9 (90.0)		12 (75.0)		8 (18.6)		—	
	ECV	0 (0)		2 (12.5)		8 (18.6)		—	
	Turning a breech baby	1 (10.0)		1 (6.3)		22 (51.2)		—	
	Fetal version	0 (0)		0 (0)		3 (7.0)		—	
	Turning baby	0 (0)		1 (6.3)		0 (0)		—	

^a^No statistical analyses were performed for these variables since there was no relevance for the aim of this research.

^b^View ratio: number of views/days on platform.

^c^Like ratio: likes×100/(likes+dislikes).

^d^Video power index: (like ratio×view ratio)/100.

^e^Usefulness score: total ECV RCOG–specific score+total mDISCERN score–misinformation; the score has a maximum of 30 points and videos are accordingly classified as not useful (<9 points), slightly useful (9-13 points), and useful (>13 points).

^f^RCOG: Royal College of Obstetricians & Gynecologists.

^g^mDISCERN: modified DISCERN score [24]; a validated tool that measures the reliability of patient information concerning treatment choice.

### Source of Upload

The included videos were evenly spread across the upload categories. Three videos could not be categorized in the predefined subgroups and were thus included in the “other” group. These videos included a news channel and a government organization, whereas one source was miscellaneous. Characteristics of the source of upload are shown in Table 3. Childbirth attendants appear to be the most influential sources on this topic, scoring the highest on audience engagement (view ratio, like ratio, and VPI). Excluding the “other” group, paramedics scored the lowest on audience engagement factors.

Usefulness scores were calculated for each category. Educational channels scored the highest, followed by health care professionals. Childbirth attendants scored the lowest on the total usefulness score, with vloggers scoring only slightly above. There was a statistically significant difference in usefulness scores according to the source of upload (*P*<.001).

When comparing the mean score for each ECV RCOG–specific score statement, “putting pressure on the mothers’ belly” and “aim for vaginal birth” scored the highest, whereas the statements “50% chance of succeeding” and “chance of turning back” scored the lowest. The questions “Are the aims clear and achieved?” and “Is the information presented balanced and unbiased?” had the highest mean scores of the four mDISCERN statements. Points that were deducted most often came from the misinformation categories *proven risks* and *unproven procedure*.

**Table 3:** Characteristics of the YouTube videos on external cephalic version (ECV) according to the source of upload.

Characteristics			Childbirth attendant (n=13, 18.6%), mean (SD)		Paramedic (n=6, 8.6%), mean (SD)		Vlogger (n=19, 27.1%), mean (SD)		Health care professional (n=12, 17.1%), mean (SD)		Educational channel (n=17, 24.3%), mean (SD)		*P* value
Years since upload			3.6 (2.9)		4.8 (2.4)		4.4 (3.4)		5.1 (3.6)		4.0 (2.8)		—^a^
Duration (minutes)			12.6 (13.7)		4.3 (2.7)		9.9 (5.2)		5.1 (2.7)		11.2 (11.2)		—
**Audience engagement**													
	View ratio^b^	218.5 (348.6)		40.5 (33.6)		242.9 (730.2)		242.5 (398.1)		204.7 (510.8)		.11	
	Like ratio^c^	95.8 (4.9)		94.5 (5.0)		9.4 (6.9)		88.2 (8.1)		84.8 (23.3)		.23	
	Video power index^d^	250.5 (343.4)		45.5 (32.4)		211.3 (617.8)		226.0 (357.7)		167.9 (413.7)		.13	
**Usefulness score^e^**													
	Total usefulness score	2.9 (2.5)		7.3 (2.3)		4.8 (2.5)		10.9 (6.8)		12.0 (5.5)		<.001	
	ECV RCOG^f^–specific score points awarded	1.4 (2.6)		4.8 (1.7)		4.0 (2.2)		6.3 (5.4)		7.7 (4.3)		<.001	
	mDISCERN^g^ points awarded	2.5 (1.4)		3.1 (0.7)		1.4 (0.6)		5.0 (1.9)		5.1 (1.7)		<.001	
	Points deducted for misinformation	1.0 (1.3)		0.6 (0.7)		0.5 (0.7)		0.5 (0.9)		0.8 (1.1)		.74	

^a^No statistical analyses were performed for these variables since there was no relevance for the aim of this research.

^b^View ratio: number of views/days on platform.

^c^Like ratio: (likes×100)/(likes+dislikes).

^d^Video power index: (like ratio×view ratio)/100.

^e^Usefulness score: total ECV RCOG–specific score+total mDISCERN score–misinformation; the score has a maximum of 30 points and videos are accordingly classified as not useful (<9 points), slightly useful (9-13 points), and useful (>13 points).

^f^RCOG: Royal College of Obstetricians & Gynecologists.

^g^mDISCERN: modified DISCERN score [24]; a validated tool that measures the reliability of patient information concerning treatment choice.

### Medical Versus Nonmedical Videos

All evaluated videos were divided into medical (n=32) and nonmedical (n=38) videos and their characteristics are summarized in Table 4. Overall, nonmedical videos had higher audience engagement than medical videos.

Approximately one-quarter of the medical videos were considered useful, whereas only 5.3% of the nonmedical videos were considered useful. Almost all of the nonmedical videos were considered not useful, whereas 34.4% of the medical videos were considered not useful. The medical videos that were categorized as not useful scored low on the ECV RCOG–specific score and average on the mDISCERN. Moreover, over 75% of medical videos were uploaded by educational channels and health care professionals. The majority of nonmedical videos were uploaded by childbirth attendants and vloggers. Two-thirds of the medical videos were found with the search term “external cephalic version,” whereas most of the nonmedical videos were found with the search terms “turning a breech baby” and “ECV.”

**Table 4:** Characteristics of videos on YouTube about external cephalic version (ECV) based on a medical or nonmedical source of upload.

Characteristics		Medical videos (n=32, 45.7%)	Nonmedical videos (n=38, 54.3%)	*P* value	
Years since upload, mean (SD)		4.8 (3.2)	4.4 (3.3)	—^a^	
Duration (minutes), mean (SD)		7.7 (9.2)	10.0 (9.2)	—	
**Audience engagement, mean (SD)**					
	View ratio^b^	206.7 (448.4)	237.4 (571.0)	.33	
	Like ratio^c^	91 (9)	89.75 (16.44)	>.99	
	Video power index^d^	184.5 (380.9)	218.8 (496.7)	.42	
**Usefulness score^e^, mean (SD)**					
	Total usefulness score	10.7 (4.3)	5.0 (4.3)	<.001	
	ECV RCOG^f^ score points awarded	6.5 (3.5)	3.4 (3.5)	<.001	
	mDISCERN^g^ points awarded	4.8 (1.5)	2.2 (1.5)	<.001	
	Points deducted for misinformation	0.6 (1.0)	0.7 (1.0)	.89	
**Usefulness category, n (%)**					<.001
	Useful	8 (25.0)	2 (5.3)		
	Slightly useful	13 (40.6)	3 (7.9)		
	Not useful	11 (34.4)	33 (86.8)		
**Source of upload, n (%)**					<.001
	Health care professional	12 (37.5)	0 (0)		
	Paramedic	6 (18.0)	0 (0)		
	Vlogger	0 (0)	19 (50.0)		
	Education channel	13 (40.6)	4 (10.5)		
	Childbirth attendants	0 (0)	13 (34.2)		
	Other	1 (3.1)	2 (5.3)		
**Search term, n (%)**					.01
	External cephalic version	20 (62.5)	10 (26.3)		
	ECV	3 (9.4)	7 (18.4)		
	Turning a breech baby	8 (25.0)	15 (39.5)		
	Fetal version	1 (3.1)	0 (0)		
	Turning baby	0 (0)	5 (13.2)		

^a^No statistical analyses were performed for these variables since there was no relevance for the aim of this research.

^b^View ratio: number of views/days on platform.

^c^Like ratio: (likes×100)/(likes+dislikes).

^d^Video power index: (like ratio×view ratio)/100.

^e^Usefulness score: total ECV RCOG score+total mDISCERN score–misinformation; the score has a maximum of 30 points and videos are accordingly classified as not useful (<9 points), slightly useful (9-13 points), and useful (>13 points).

^f^RCOG: Royal College of Obstetricians & Gynecologists.

^g^mDISCERN: modified DISCERN score [24]; a validated tool that measures the reliability of patient information concerning treatment choice.

### Framing of the Videos

Lastly, we evaluated whether videos uploaded by vloggers were encouraging or discouraging of ECV. As shown in Table 5, ECV was not mentioned in 4 videos in favor of other methods to attempt to turn the baby from a breech to cephalic position. Almost all vloggers (12/15, 80%) encouraged ECV to their followers. Videos encouraging ECV had higher audience engagement compared to videos discouraging ECV, with a particularly large difference in the VPI (313.0 vs 17.2). The like ratio did not differ between the groups (mean 92.6 vs 93.6, *P*=.47). Three-quarters of the encouraging videos were uploaded by vloggers who experienced a successful ECV.

**Table 5:** Characteristics of videos on YouTube about external cephalic version (ECV) uploaded by vloggers, categorized according to whether they discouraged or encouraged ECV (N=15).

Characteristics	Discouraging	Encouraging
Videos, n (%)	3 (15.8)	12 (63.2)
View ratio^a^, mean (SD)	19.0 (13)	359.7 (898.0)
Like ratio^b^, mean (SD)	93.6 (4.7)	92.6 (5.8)
VPI^c^, mean (SD)	17.2 (11.2)	313.0 (758.9)
Experienced successful ECV, n (%)	0 (0)	9 (75)
Experienced unsuccessful ECV, n (%)	3 (100)	3 (25)

^a^View ratio: number of views/days on platform.

^b^Like ratio: (likes×100)/(likes+dislikes).

^c^VPI: video power index; (like ratio×view ratio)/100.

## Discussion

### Principal Results

In this study, according to the accuracy of medical information and content quality, only a minority of videos related to ECV on YouTube were found to be useful. Strikingly, the group with the highest audience engagement scored the lowest on usefulness and the groups with lower audience engagement had higher usefulness scores. The presence of misinformation was low. Importantly, vloggers, the group with the highest audience engagement and the lowest usefulness score, largely encouraged their audience to opt for ECV.

### Comparison With Prior Work

In general, our findings are consistent with previous research evaluating the quality of health-related subjects on YouTube [16,20,27,28]. In a study performed by Lee et al [27] in 2014 evaluating YouTube videos on cholecystolithiasis, 56.5% of the videos were classified as misleading and only 13.8% were classified as useful or very useful. Similarly, we found that very useful videos about ECV had a significantly lower mean view average than that of the misleading videos. In a study on content analysis of infertility-related videos on YouTube performed in 2018, Kelly-Hendrick et al [29] found that videos delivered from a personal point of view had significantly more likes than informational-educational videos. These findings suggest that people who are looking for videos on health-related subjects appear to rely more on personal experiences and information from their peers. This results in overexposure to scientifically inaccurate videos, although these are not necessarily misleading and may even encourage pregnant people to opt for ECV.

Among the search terms, medical videos with accurate information were largely identified when using medical terms, whereas nonmedical videos were identified with nonmedical terminology in the YouTube search.

As a result of an aging YouTube collection along with continuously developing ECV guidelines, medical videos on YouTube also contain misinformation. For example, medical videos contained misinformation on risks by stating that a previous cesarean section is a contraindication for ECV, whereas the RCOG guideline states that ECV is safe and successful in women with one previous cesarean section [5]. YouTube does not filter these outdated videos, underpinning that responsible information providers should also have a plan for updating or removing outdated videos.

In the evaluated videos, alternatives for ECV to encourage spontaneous fetal version were often mentioned. The childbirth attendants advised yoga postures, warm and cold compresses, and acupuncture in combination with misbutton and chiropractic treatment to support a baby in turning to be in a cephalic position. This is in contradiction with the RCOG guideline statement that there is no evidence that postural management alone promotes spontaneous version to a cephalic presentation [5]. People who refuse ECV might be interested in alternative methods for spontaneous version. Therefore, future research should focus on the association between spontaneous version aided by chiropractors and postural management in combination with moxibustion and acupuncture.

The importance of appropriate and encouraging information about ECV for breech presentation is evident, since ECV is of great significance to reduce the CD rate [30]. In 2014, Vlemmix et al [9] found that less than half of pregnant people with a breech baby at term had an ECV in the Netherlands, 20%-30% of pregnant people refused an ECV, and it was estimated that approximately 4%-33% of patients were not given the option of having an ECV by their health care providers. YouTube can be valuable as a source of information for this young generation that is adept at using digital technology for communication and information gathering.

### Strengths and Limitations

This study has several strengths and limitations. First, we used the validated mDISCERN score and the customized ECV RCOG–specific score based on the RCOG guideline. However, it must be acknowledged that the partly self-developed usefulness score remains somewhat subjective due to the absence of validated tools for evaluating social media content. Although high agreement was achieved between the two video reviewers, this limitation underscores the pressing need for a standardized scoring instrument in modern research. The increasing prevalence of video content across various fields demands a reliable method of assessing video quality and content. Without a standardized and validated scoring instrument, the evaluation is prone to subjectiveness, inconsistency, and lack of scientific accuracy. Establishing and validating this instrument is essential for contemporary research to conduct meaningful studies, make informed decisions, and enhance the medical accuracy of video content. Such studies can help pave the way for more precise assessments and comparisons.

Second, the RCOG guideline was used as the up-to-date and high-quality standard, although this is not necessarily the standard in all contexts.

Third, the methods of the search may have been selective. We only used five search terms, only the first 35 results were retained, and the search was conducted in “Incognito” mode. Because a YouTube search is dynamic and dependent on algorithms, the findings may be variable based on new uploaded videos and personal algorithms.

Finally, we only searched YouTube and omitted other social media platforms that play a role as a source of information for patients with unregulated content. However, YouTube is most likely to be the platform that patients use in the search of health information, as it is the largest video-sharing website with the highest number of videos without time limits and has a user-friendly search function that does not require an account to watch videos.

### Implications and Directions for Future Research

While our study has shed light on the medical accuracy of YouTube videos about ECV, it is important to acknowledge that we did not explicitly address the potential impact of various video characteristics, including video length, characteristics of the audience, the uploader’s username, or word-of-mouth recommendations, on the usefulness scores of the videos. These factors are of great importance in understanding the dynamics of online health content consumption. For example, video length could affect viewers’ engagement and retention of information, while the uploader’s name or reputation can influence viewers’ trust in the content. YouTube adds a view when a video is watched for over 30 seconds; however, YouTube does not disclose the actual watched duration. Furthermore, individual user data are anonymized and demographic information of the viewer is only available for the uploader. Future research should explore these additional factors comprehensively, as they have the potential to significantly influence viewers’ interactions with health-related videos, ultimately impacting their overall usefulness and the quality of health information accessible on the platform.

This study highlights the compelling need for health care professionals to collaborate with social media influencers to create medically accurate videos with a broad reach and high viewership. In an era where online content holds substantial sway, leveraging the reach and engagement of influencers can significantly enhance the dissemination of credible health information and promote public awareness. Partnerships between hospitals and vloggers can bridge the gap between traditional health care communication channels and the rapidly evolving digital landscape, thereby improving health literacy and encouraging informed decision-making among the public. Balancing content popularity with medical accuracy will be a paramount challenge, requiring careful oversight to ensure that influencers and health care professionals collaboratively contribute to a well-informed and healthier society.

### Conclusions

Despite the fact that only few YouTube videos on ECV were considered to be useful, the level of misinformation on ECV in the videos was low. The mismatch between audience engagement and information accuracy was striking, although the vlogs with the highest engagement were appropriately encouraging their audience to opt for an ECV. The findings from this study provide the insight that informational videos should contain valid information along with encouraging stories from the patient perspective using nonmedical terminology to have a maximum impact. This can likely best be achieved by collaboration between medical and community stakeholders. This would generate more awareness about ECV, support pregnant people with a baby in breech presentation, and lower the chance for a high-risk delivery because of term breech presentation.

## Appendix

Multimedia Appendix 1 Flowchart of the selection of included YouTube videos. ECV: external cephalic version.

Multimedia Appendix 2 Baseline characteristics of included videos.

## Abbreviations

CD: cesarean delivery
ECV: external cephalic version
mDISCERN: modified DISCERN score
RCOG: Royal College of Obstetricians & Gynaecologists
VPI: video power index

## Data Availability

The data sets generated and analyzed during this study are available from the corresponding author on reasonable request.
