# Supplementary material for: YouTube as a Source of Patient Information on External Cephalic Version: Cross-Sectional Study
Source: JMIR Form Res. 2024 Jun 6;8:e50087. doi: 10.2196/50087 (PMC11190616; doi:10.2196/50087)
Supplement: Multimedia Appendix 1 [file formative_v8i1e50087_app1.docx]

**Multimedia appendix**

**Multimedia appendix 1:** flowchart of included YouTube videos

Videos extracted from YouTube

5 search terms

External cephalic version (n=35)

EVC (n=35)

Turning a breech baby (n=35)

Fetal version (n=35)

Turning baby (n=35)

175 videos

129 videos screened

Duplicates removed (n=46)

Videos excluded

Content not related to breech (n=55)

Language other than English (n=2)

Other (n=2)

70 videos included in review
