# Supplementary material for: YouTube as a Source of Patient Information on External Cephalic Version: Cross-Sectional Study
Source: JMIR Form Res. 2024 Jun 6;8:e50087. doi: 10.2196/50087 (PMC11190616; doi:10.2196/50087)
Supplement: Multimedia Appendix 2 [file formative_v8i1e50087_app2.docx]

**Multimedia appendix 2:** Baseline characteristics and characteristics of YouTube videos.

|  | All videos  (n=70) |  |
| --- | --- | --- |
| Years since upload (m) SD | 4.6 | 3.3 |
| Duration (minutes) (m) SD | 8.9 | 9.3 |
| View ratio^a^ (m) SD | 223.4 | 519.0 |
| Like ratio^b^ (m) SD | 90.2 | 13.6 |
| Video power index^c^ (m) SD | 203.8 | 449.8 |
| Usefulness score^d^ (m) | 7.6 | 5.6 |
| ERSS^e^ points awarded (m) SD | 4.8 | 4.2 |
| mDISCERN^f^ points awarded (m) SD | 3.4 | 2.1 |
| Points deducted (m) SD | 0.7 | 1.0 |

m=mean

n=number

SD=standard deviation

^a^View ratio = number of views/days

^b^Like ratio = like × 100/ (like + dislike)

^c^Video Power Index = like ratio × view ratio/100

^d^Usefulness score = total ERSS score + total mDISCERN score

^e^ERSS = ECV RCOG Specific score is based on the patient information web page of the Royal College of Obstetricians & Gynecologists (RCOG)

^f^mDISCERN = The modified DISCERN score is a validated tool that measures the reliability of patient information concerning treatment choice.
